# Sugar Content of Children’s Breakfast Foods in Mediterranean Diet Patterns

**DOI:** 10.3390/nu17172717

**Published:** 2025-08-22

**Authors:** Clara Guinot-Barona, Giorgia Tumino, Marta Ibor-Miguel, Carla Borrell-García, Juan-Ignacio Aura-Tormos, Esther García-Miralles, Laura Marqués-Martínez

**Affiliations:** 1 Dentistry Department, Faculty of Medicine and Health Sciences, Catholic University of Valencia San Vicente Mártir, 46001 Valencia, Spaincarla.borrell@ucv.es (C.B.-G.);; 2 Faculty of Medicine and Dentistry, University of Valencia, 46010 Valencia, Spain

**Keywords:** oral health, dental caries, diet, processed foods, breakfast, children

## Abstract

Background: Breakfast habits in Mediterranean countries often include processed products with hidden sugars, which may compromise children’s oral and general health. Objectives: This study assessed the sugar content of breakfast foods commonly consumed by children using °Brix refractometry and examined its implications for dental caries and obesity. Methods: Forty-nine breakfast food samples (processed products, homemade alternatives, and fresh fruits) were analysed using a digital °Brix refractometer to quantify soluble sugar concentrations. Comparative statistical analyses were performed to evaluate differences among food categories. Results: Processed foods consistently exhibited significantly higher °Brix values (mean ± SD: 14.1 ± 4.9), reflecting greater levels of extrinsic sugars, compared with homemade preparations (10.9 ± 1.1) and fresh fruits (10.7 ± 5.2) (*p* < 0.01). Processed items contained on average 25% more sugar than the other categories. Fresh fruits and homemade options demonstrated moderate °Brix levels, with no added sugars, whereas processed products—despite some being marketed as “no added sugars”—frequently contained substantial sugar content. Conclusions: The findings highlight the urgent need for educational strategies and clearer labelling to reduce sugar intake during childhood breakfasts. Promoting natural and homemade alternatives could be a key preventive approach to lowering the risk of dental caries, obesity, and other diet-related conditions.

## 1. Introduction

Breakfast is widely acknowledged as a fundamental component of a healthy diet, particularly for children, as it provides essential nutrients and energy following an overnight fast. Its regular consumption has been associated with improved cognitive development, academic performance, and overall nutritional status in school-aged children [1,2]. In addition to supporting cognitive and physical growth, breakfast has been linked to better metabolic regulation, including appetite control and reduced total energy intake throughout the day [3].

However, increasing attention has been paid to the nutritional quality of children’s breakfast foods. Many processed products, frequently consumed in the morning, are rich in added sugars and have been associated with two major public health concerns: dental caries and childhood obesity [4,5]. The excessive consumption of free sugars is strongly linked to cariogenic risk due to their capacity to lower oral pH and promote acidogenic bacterial growth within dental biofilm [6]. Moreover, high-sugar diets contribute to an energy imbalance and fat accumulation, increasing the risk of overweight and metabolic disturbances [7]. Sugar-sweetened beverages, often marketed directly to children, have been identified as a significant source of free sugars and are associated with an excessive caloric intake and adverse health outcomes [8,9,10].

In Mediterranean countries, children’s breakfasts traditionally include dairy products, cereals, and fresh fruits. Nevertheless, contemporary dietary habits show a growing trend towards industrially processed options, many of which contain substantial extrinsic sugars [11,12]. These changes not only affect dietary quality but also contribute to the early onset of diet-related diseases, including dental caries and obesity. In response, recent public health strategies have emphasised the importance of promoting healthier breakfast alternatives as a preventive approach to both oral and systemic health [2,13,14].

Among the responses to these concerns is the reformulation of food products using sugar substitutes, such as xylitol, which may reduce the cariogenic potential [15]. However, evidence regarding the long-term efficacy and safety of these non-nutritive sweeteners in paediatric populations remains limited [16]. Additionally, front-of-pack nutrition labelling (FOPL), including interpretive schemes like Nutri-Score, has been introduced to guide consumers toward healthier choices, though its real-world impact remains inconsistent [17,18].

Reliable and objective methods for quantifying the sugar content in children’s foods are needed in order to inform both consumers and policy efforts. The °Brix refractometer is a widely used instrument in the food industry that measures the concentration of soluble solids—mainly sugars—in liquid and semi-liquid samples. Brix refractometry is a rapid, non-destructive, and cost-effective method to estimate the total soluble solids in foods, which correlate strongly with the sugar content. Unlike chromatographic or enzymatic assays, °Brix measurement requires minimal sample preparation and allows immediate in situ assessment, making it particularly suitable for screening a large variety of food products in field studies and public health contexts. This method offers an accurate, reproducible, and cost-effective means of assessing sweetness levels in commercially available products [17,19].

Therefore, the aim of this study is to evaluate the sugar content of breakfast foods commonly consumed by children in Mediterranean countries using °Brix refractometry, and to analyse its potential association with the risk of dental caries and childhood overweight.

## 2. Materials and Methods

A laboratory-based analysis research study was conducted in January 2024 at the Catholic University of Valencia San Vicente Mártir to evaluate the sugar content of breakfast foods commonly consumed by children. This study aimed to understand the potential implications of these foods for dental caries and other health-related issues. A total of 49 food samples, representing a broad spectrum of products routinely consumed by preschool and school-aged children in the Mediterranean region, were analysed. The selection criteria for these foods included registered brands available in supermarkets, liquid-based products, and freshly squeezed seasonal fruits.

Processed foods were defined as commercially packaged products containing added sugars, sweeteners, or other industrial ingredients as per the NOVA classification system (categories 3–4). Homemade foods were defined as items prepared entirely from raw or minimally processed ingredients within the household, without the addition of commercial sweeteners or pre-packaged mixes. Fresh fruits were unprocessed and served raw, without any added ingredients.

A digital Brix refractometer (PAL-1 model, ATAGO Co., Tokyo, Japan) was used to measure °Brix values. The refractometer, a precision instrument extensively used in the food industry to measure sugar concentrations, was employed for the analysis. This device quantifies the refraction of light through liquid samples, providing a quantitative measure of dissolved solids, primarily sugars, expressed in °Brix. Brix measurements are essential for assessing the sweetness, taste, and consistency of food products, thereby serving as a critical tool for quality control in products such as fruits, juices, syrups, and honey.

Brix-to-sugar conversion assumed 1°Brix ≈ 1 g sugar per 100 g solution, consistent with ISO 2173:2003 for fruit products (ISO 2173:2003) [20].

Prior to analysis, samples from registered brands, including yogurts, jars, pouches, juices, and seasonal fruits, were collected, ensuring adherence to all inclusion and exclusion criteria. For homemade samples, proportions indicated on packaging labels were used to prepare 10 g mixtures, with weights accurately measured using laboratory scales. The samples were then subjected to refractometry, involving meticulous steps: cleaning the refractometer prism, applying 1–2 drops of the sample while avoiding air bubbles, closing the device to evenly distribute the liquid, observing under appropriate lighting, calibrating the scale for precision, and thoroughly cleaning the prism after each measurement to prevent contamination [17]. The °Brix refractometer was calibrated before each product measurement using distilled water (0 °Brix) and a 10% sucrose control solution to verify accuracy. Calibration was repeated if deviations greater than ±0.1 °Brix were detected.

The study analysed a total of 49 breakfast food samples, distributed across three categories: processed products (*n* = 30), homemade alternatives (*n* = 7), and fresh seasonal fruits (*n* = 12).

Statistical analyses were performed using IBM SPSS Statistics 22 (α = 0.05). Descriptive statistics calculated included the sample size (n) and mean ± standard deviation values. Inferential analysis involved a one-way ANOVA to assess differences among the three food groups, followed by Bonferroni post hoc tests for pairwise comparisons. This comprehensive and methodical approach ensured the reliability and validity of the data collected, providing a robust basis for evaluating the sugar levels in children’s breakfast foods and their health implications.

Ethical review was waived as this study analysed commercial products and did not involve human participants or animals.

## 3. Results

The quantitative analysis of the 49 breakfast food samples revealed significant variations in sugar content, expressed in °Brix, across processed, homemade, and fresh food categories. These differences have direct implications for paediatric oral health and obesity risk. The findings are summarised below.

### 3.1. Sugar Content in Processed Foods

Processed breakfast products exhibited the highest °Brix values, ranging from 2.0 to 19.0°Brix (Table 1). Approximately 68% of these items contained added sugars, confirming their significant contribution to children’s sugar intake.

Particularly elevated °Brix values were observed in sweetened yogurts, such as Danonino^®^ banana-strawberry (10.0° Brix), and fruit pouches, with the Nestlé^®^ 4-fruit variant reaching 17.5° Brix. Notably, products labeled “no added sugars,” including Pascual^®^ pineapple yogurt (2.0° Brix), still presented measurable sugar levels, likely due to natural sweeteners or intrinsic sugars. The presence of alternative sweeteners raises concerns about potential long-term health implications.

Vegetable-based baby jars, such as Gerber^®^ peas and potatoes (6.9° Brix), exhibited comparatively lower sugar levels. However, five out of six samples in this category still contained added sugars, reinforcing the need for a cautious interpretation of “vegetable” product labels.

### 3.2. Sugar Content in Homemade Alternatives

Homemade preparations demonstrated a narrower °Brix range, between 9.4 and 13.0, with 92% of samples free from added sugars (Table 2). Only the homemade mixture of milk with cocoa powder (12.3° Brix) contained added sugar.

The remaining samples, primarily composed of blended fruits and milk, achieved comparable sweetness to processed foods without artificial sweeteners. For instance, a banana–strawberry milkshake recorded 9.4° Brix, and a more complex blend of banana, apple, and peach reached 12.0° Brix. These findings suggest that natural ingredients can replicate the palatability of industrial products while minimising health risks.

### 3.3. Sugar Content in Fresh Seasonal Fruits

Fresh fruits exhibited a wide °Brix range, from 2.0 to 19.0 (Table 3), reflecting their natural sugar content. Black grapes (19.0° Brix) and apples (16.0° Brix) had the highest values, while lemons (2.0° Brix) had the lowest.

### 3.4. Statistical Analysis

**Figure 1:**
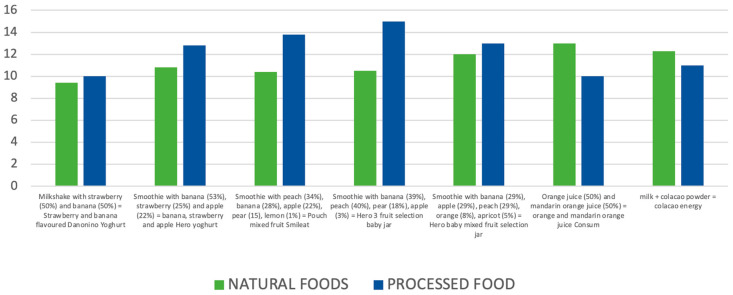
Average °Brix values in processed, homemade, and fresh breakfast foods. Processed products show significantly higher sugar concentrations (*p* < 0.01). Error bars indicate standard deviations.

**Figure 2:**
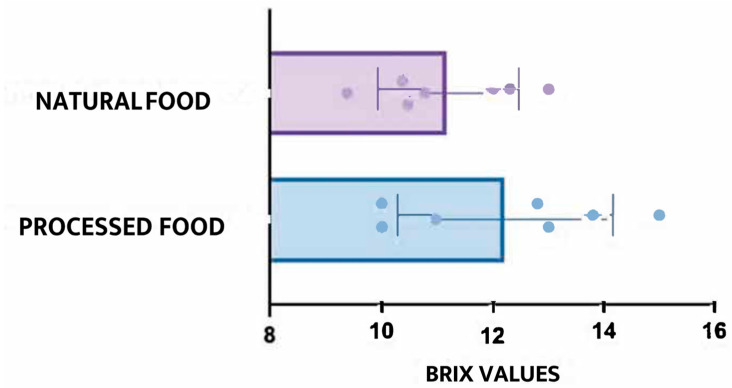
Distribution of °Brix values across food categories. Boxplots highlight greater variability in processed foods.

The comparative statistical analysis revealed several significant findings. Processed foods exhibited, on average, 25% higher °Brix values than their homemade or fresh counterparts, as determined by a paired *t*-test (*t* (48) = 4.67, *p* = 0.003). Additionally, a greater variability in sugar concentration was identified among processed products, as evidenced by Levene’s test for equality of variances (F = 8.3, *p* = 0.004). In contrast, no statistically significant differences were observed between homemade foods and fresh fruits, with the Pearson correlation indicating a weak and non-significant association (r = 0.20, *p* = 0.35). These results are visually represented in Figure 1 and Figure 2. Figure 1 displays the average °Brix values for each group, whereas Figure 2 shows the distribution of values through boxplots, emphasising the wider range of values observed in processed items.

## 4. Discussion

Breakfast plays a pivotal role in shaping children’s nutritional patterns, metabolic regulation, and oral health outcomes. In Mediterranean countries such as Spain, Italy, and Greece, breakfast is traditionally consumed at home; however, the increasing market penetration of industrially processed foods has altered this pattern, often leading to higher free sugar intake in children [21,22]. Our findings of significantly higher °Brix values in processed breakfast products compared to homemade and fresh fruit preparations are consistent with recent surveys conducted in Italy [23], Greece [24], and Portugal [25], which report mean °Brix values between 13–15 in commercially packaged children’s foods. Similar patterns have been observed globally, including in the UK [26], Australia [27], and Brazil [28], suggesting that elevated sugar content in packaged foods for children is a widespread phenomenon.

While WHO guidelines recommend limiting free sugar intake to less than 10% of total energy, translating these recommendations into effective national policies remains challenging. In Spain and other Mediterranean countries, barriers include the inconsistent regulation of marketing claims, limited enforcement of sugar thresholds in processed foods, and cultural dietary habits that increasingly incorporate packaged snacks and beverages into children’s breakfasts [29].

Although °Brix refractometry is widely recognised as a cost-effective and reproducible method for estimating the sugar content in foods and has been used in WHO-supported nutrition monitoring projects [24], it is not an officially endorsed WHO standard for sugar surveillance. Its ease of use makes it suitable for large-scale screening, particularly in public health and school-based interventions, but the results should be interpreted alongside more specific analytical techniques such as high-performance liquid chromatography when precise sugar profiling is required.

Several limitations should be acknowledged. First, °Brix refractometry measures total soluble solids, not exclusively sugars; thus, other soluble compounds may slightly influence readings. Second, the cross-sectional design of this study precludes causal inference regarding the relationship between sugar content and health outcomes. Third, brand availability at the time of sampling and the seasonality of fresh fruits may limit the generalizability of our findings to other regions or periods. Finally, although the study included a range of product types, the relatively small number of homemade samples reflects a lower household participation and may affect comparisons.

Overall, our results highlight the need for targeted nutritional education and stricter labeling regulations to reduce children’s exposure to excessive free sugars at breakfast. A comparative °Brix analysis offers a practical tool for the ongoing surveillance of sugar content in children’s foods, particularly when combined with consumer education strategies and policy enforcement aimed at improving the quality of breakfast options in Mediterranean and global contexts [30,31,32,33].

## 5. Conclusions

This study found that processed breakfast foods for children contained significantly higher °Brix values—indicating greater levels of extrinsic sugars—compared to homemade preparations and fresh fruits. These findings are consistent with evidence from other Mediterranean and international studies and highlight the widespread presence of added sugars in packaged products marketed to children.

While °Brix refractometry cannot distinguish between types of soluble solids, its practicality and reproducibility make it a useful tool for rapid screening of sugar content in public health nutrition monitoring. The results of this study support the need for continued surveillance, clearer product labeling, and educational initiatives aimed at reducing children’s sugar intake, particularly at breakfast. Further research, including longitudinal studies and detailed compositional analyses, is warranted to strengthen the evidence base and guide effective public health interventions.

## Figures and Tables

**Table 1:** Brix degree and presence of extrinsic sugars in processed breakfast foods commonly consumed by children. Higher °Brix values reflect elevated levels of soluble sugars. The presence of extrinsic sugars (✔) indicates added sugars beyond those naturally occurring in ingredients, ✘= no extrinsic sugars added.

Processed Food	GRADE BRIX (% Sugar in 100 g Solution)	Extrinsic Sugars
Danonino ^®^gluten-free banana- and strawberry-flavoured sweetened yoghurt	10° Brix	3/11
Danonino ^®^semi-skimmed drinking sweetened yoghurt with strawberry, calcium, and vitamin D	9.8° Brix	2/11
Banana, strawberry, and apple sweetened yoghurt Hero^®^	12.8° Brix	0
Pascual ^®^strawberry sweetened yoghurt with no added sugars	3.8° Brix	6/10
Sweetened yoghurt with pineapple and no added sugar Pascual^®^	2.0° Brix	2/7
Strawberry-flavoured sweetened kids’ smoothie Consum^®^	10° Brix	2/8
Assorted fruit sweetened pouch Smileat^®^	13.8° Brix	**✘**
My Hero^®^ Snack sweetened Pouch	12.5° Brix	**✘**
Nestlé Yogolino ^®^ sweetened Strawberry Apple Pouch	8.6° Brix	**✘**
Nestlé ^®^Pouch sweetened 4 fruits	17.5° Brix	**✘**
Hero Baby ^®^ Jar sweetened selection of three fruits	15.0° Brix	**✘**
Hero Baby ^®^Jar selection of assorted fruits sweetened	13.0° Brix	**✘**
Be plus ^®^fruit jar sweetened	16° Brix	5/6
Gerber^®^ Peas and Potato Jars sweetened	6.9° Brix	**✘**
Vegetable and veal Gerber^®^	5.9° Brix	**✘**
Chicken and rice Be plus^®^	10.0° Brix	**✘**
Hero Baby ^®^Beef Stew Jar	8.8° Brix	**✘**
Hero Baby ^®^Vegetable Stew Jar	8.7° Brix	**✘**
Don Simon^®^ Pineapple Juice	12.0° Brix	**✘**
Nestlé Junior ^®^Growing-Up sweetened Milk	10.5° Brix	**✘**
Asturiana^®^ semi-skimmed milk	10.2° Brix	**✘**
Nestlé ^®^Native Growth Milk sweetened	6.8° Brix	**✘**
Tropical kids sweetened drink with fruit juice and tropical Consum^®^	7.6° Brix	2/5
Orange and Mandarin sweetened Juice Consum^®^	10° Brix	1/3
Kids’ sweetened drink with fruit and milk Mediterranean Consum^®^	7.7° Brix	2/5
Colacao ^®^energy drink sweetened	11.0° Brix	2/9
Yogolino sweetened custard Nestlé^®^	13.0° Brix	2/7
Yogolino Nestlé ^®^Cocoa sweetened	13.6° Brix	2/5
Nestlé Nesquik^®^ Sweetened chocolate Milkshake	10.5° Brix	3/9
Sweetened drink with cereals and cocoa Puleva^®^	12° Brix	3/13
Nesquik^®^ Chocolate Petit Yoghurt sweetened	19.0° Brix	2/10

**Table 2:** Brix values and extrinsic sugar content in homemade alternatives replicating commercial breakfast products. Most samples exhibit moderate sugar concentrations derived from natural ingredients, with minimal addition of extrinsic sugars (**✘**= no extrinsic sugars added).

Home-Made Foods from the Composition of Some Processed Foods	Grade Brix	Extrinsic Sugars
Milk + powdered cocoa powder (homemade)	12.3° Brix	1/6
Homemade milkshake with strawberry (50%) and banana (50%)	9.4° Brix	**✘**
Homemade milkshake with banana (53%), strawberry (25%) and apple (22%)	10.8° Brix	**✘**
Homemade milkshake with peach (34%), banana (28%), apple (22%), pear (15%), and lemon (1%)	10.4° Brix	**✘**
Homemade milkshake with banana (39%), peach (40%), pear (18%), and apple (3%)	10.5° Brix	**✘**
Homemade milkshake with banana (29%), apple (29%), peach (29%), orange (8%), and apricot (5%)	12.0° Brix	**✘**
Homemade orange juice (50%) and mandarin juice (50%)	13.0° Brix	**✘**

**Table 3:** Natural sugar content in fresh seasonal fruits measured by Brix degree. Although some fruits exhibit high °Brix values, all samples are free from added sugars (**✘** = no extrinsic sugars added).

Fresh Seasonal Fruit	Grade Brix	Extrinsic Sugars
Black Grape	19.0° Brix	**✘**
Banana	9.0° Brix	**✘**
Mandarins	13.0° Brix	**✘**
Oranges	14.3° Brix	**✘**
Apple	16.0° Brix	**✘**
Lemon	2.0° Brix	**✘**
Peach	10.0° Brix	**✘**
Strawberry	10.0° Brix	**✘**
Pear	14.0° Brix	**✘**
Apricot	12.0° Brix	**✘**
Pineapple	14.0° Brix	**✘**

## Data Availability

The data presented in this study are available upon request from the corresponding author. The data are not publicly available due to the nature of the commercial product analysis.
